# Sensitivity of diagnostic methods for *Mansonella ozzardi* microfilariae detection in the Brazilian Amazon Region

**DOI:** 10.1590/0074-02760170321

**Published:** 2018-03

**Authors:** Jansen Fernandes Medeiros, Gilberto Fontes, Vilma Lopes do Nascimento, Moreno Rodrigues, Jacob Cohen, Edmar Vaz de Andrade, Felipe Arley Costa Pessoa, Marilaine Martins

**Affiliations:** 1 Fundação Oswaldo Cruz-Fiocruz, Laboratório de Entomologia, Porto Velho, RO, Brasil; 2 Universidade Federal de São João Del-Rei, Divinópolis, MG, Brasil; 3 Fundação de Medicina Tropical Dr Heitor Vieira Dourado, Gerência de Parasitologia, Manaus, AM, Brasil; 4 Universidade Federal de Rondônia, Laboratório de Bioecologia de Insetos, Porto Velho, RO, Brasil; 5 Universidade Federal do Amazonas, Departamento de Oftalmologia, Manaus, AM, Brasil; 6 Universidade Federal do Amazonas, Centro de Apoio Multidisciplinar, Manaus, AM, Brasil; 7 Fundação Oswaldo Cruz-Fiocruz, Centro de Pesquisa Leônidas e Maria Deane, Laboratório de Ecologia de Doenças Transmissíveis na Amazônia, Manaus, AM, Brasil

**Keywords:** Mansonella ozzardi, mansonelliasis, Solimões River, Bayesian model

## Abstract

**BACKGROUND:**

The human filarial worm *Mansonella ozzardi* is highly endemic in the large tributaries of the Amazon River. This infection is still highly neglected and can be falsely negative when microfilariae levels are low.

**OBJECTIVES:**

This study investigated the frequency of individuals with *M. ozzardi* in riverine communities in Coari municipality, Brazilian Amazon.

**METHODS:**

Different diagnostic methods including polymerase chain reaction (PCR), blood polycarbonate membrane filtration (PCMF), Knott's method (Knott), digital thick blood smears (DTBS) and venous thick blood smears (VTBS) were used to compare sensitivity and specificity among the methods. Data were analysed using PCMF and Bayesian latent class models (BLCM) as the gold standard. We used BLCM to calculate the prevalence of mansonelliasis based on the results of five diagnostic methods.

**FINDINGS:**

The prevalence of mansonelliasis was 35.4% by PCMF and 30.1% by BLCM. PCR and Knott methods both possessed high sensitivity. Sensitivity relative to PCMF was 98.5% [95% confidence interval (CI): 92.0 - 99.7] for PCR and 83.5% (95% CI: 72.9 - 90.5) for Knott. Sensitivity derived by BLCM was 100% (95% CI 93.7 - 100) for PCMF, 100% (95% CI: 93.7 - 100) for PCR and 98.3% (95% CI: 90.6 - 99.9) for Knott. The odds ratio of being diagnosed as microfilaremic increased with age but did not differ between genders. Microfilariae loads were higher in subjects aged 30 - 45 and 45 - 60 years.

**MAIN CONCLUSIONS:**

PCMF and PCR were the best methods to assess the prevalence of mansonelliasis in our samples. As such, using these methods could lead to higher prevalence of mansonelliasis in this region than the most commonly used method (i.e., thick blood smears).

The species *Mansonella ozzardi* is a filarial nematode found exclusively in the neotropical region that extends from southern Mexico to northwestern Argentina, and includes the Caribbean Islands. *M. ozzardi* causes mansonelliasis. In Brazil, this parasite is highly prevalent in riverine and indigenous communities in Amazonas state, particularly in the regions of the Solimões, Purus and Negro rivers and their tributaries (Lacerda & Rachou 1956, Batista et al. 1960a, Medeiros et al. 2008, 2009, 2011, Martins et al. 2010). The filariasis caused by *M*. *ozzardi* has a symptomatology that is not well defined. In some cases this filariasis can be confused with malaria, especially in areas where the two diseases overlap (Martins et al. 2010). Symptoms include fever, chills, headache and joint pain, and the disease may be associated with ocular damage with or without corneal lesions (Batista et al. 1960b, Cohen et al. 2008, Vianna et al. 2012).

The oligosymptomatic nature of infection, negligence of treatment and a high incidence of vector bites have contributed to the high prevalence rate of this filariasis (Lacerda & Rachou 1956, Medeiros et al. 2009, 2011). Lacerda and Rachou (1956) recorded a 10.0% prevalence rate in riverine communities in Coari municipality, and a prevalence rate of 7.1 to 17.8% in Tefé municipality; both are located in the middle Solimões River region. Recent studies have reported a prevalence rate of 18.4% in Coari and 16.3% in Tefé (Martins et al. 2010, Medeiros et al. 2014). In addition, the epidemiological profile of mansonelliasis has changed: mansonelliasis has spread from rural to urban environments, and is now an emerging disease in mid-size cities in Amazonas state (Martins et al. 2010).

The thick blood smear has been the most common diagnostic method used in mansonelliasis studies conducted in Amazonas state (e.g., Batista et al. 1960a, Medeiros et al. 2008, 2009, 2011, Martins et al. 2010). Other methods, such as Knott's method (Knott) and polycarbonate membrane blood filtration (PCMF), have been used for diagnosis in a few epidemiological surveys (Moraes 1958, Batista et al. 1960a, Adami et al. 2014, Basano et al. 2016), and have also been used to monitor the effects of ivermectin in the treatment of microfilaremia (Basano et al. 2014). Medeiros et al. (2015) showed that overall *M. ozzardi* prevalence estimates based on polymerase chain reaction (PCR) from venous blood or the FTA^®^ card were 1.5 to 1.8 times higher than estimates made using microscopy to examine peripheral finger-prick blood samples.

The objective of this study was to determine the frequency of microfilaremics in riverine communities in Coari municipality, and to assess the sensitivity of different diagnostic methods used for the detection of *M. ozzardi* microfilariae.

## MATERIALS AND METHODS

**Fig. 1:**
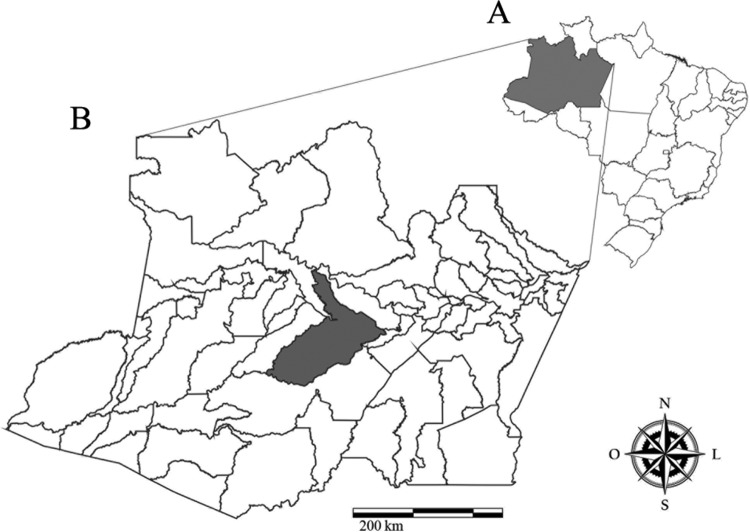
(A) map of Brazil with emphasis on the state of Amazonas. (B) Location of the study area, municipality of Coari, Amazonas state, Brazil.

*Study area* - This study was conducted in riverine communities in the municipality of Coari (4°05'S 63°08'W), Amazonas state, Brazil (Fig. 1). Coari is located in the middle Solimões River in southwestern Amazon, about 450 km from Manaus. The survey was conducted in October 2010. At the time, Coari had an estimated population of 75, 965 [urban population = 49, 638 (65.4%), rural = 26, 271 (34.6%)] with the population estimated to increase to 83,078 by 2015 (IBGE 2010). The survey was conducted by means of a convenience sample that included 189 residents (aged 6 to 77 years) of five small communities along the Solimões River. The study was approved by the Ethical Committee of the Fundação de Medicina Tropical Heitor Vieira Dourado (Protocol no. 2283/04). Informed written consent was obtained from all adults and from the parents or guardians of all children less than 18 years of age.

*Laboratory methods* - Blood samples were collected by digital puncture for diagnosis using thick blood smears and by venous puncture for diagnosis using Knott, PCMF, PCR and venous thick blood smears (VTBS).

*Digital thick blood smears (DTBS) and VTBS* - Three drops of blood (approximately 80 μL) were collected on a glass slide, spread out and dried at room temperature. After 10 h, the thick blood smears were lysed with water for 10 min, fixed with methanol and stained with Giemsa.

*PCMF of blood* - One millilitre of venous blood from each patient was diluted in 0.9% physiological saline solution and filtered in a polycarbonate membrane with pores measuring 3 μm in diameter. The membranes were then fixed with methanol and placed on Giemsa-stained glass slides. PCMF was the gold standard method in this study, because this technique has demonstrated higher sensitivity for *M. ozzardi* diagnosis (Botto et al. 1983, Vera et al. 2011, Basano et al. 2016).

*Knott* - One milliliter of venous blood from each patient was diluted in 9 mL of 2% formalin and centrifuged at 2,000 rpm for 10 min. The supernatant was removed and the pellet washed with 2% formalin. The pellet was placed on a slide, fixed with methanol and stained with Giemsa. All slides were observed under light microscopy (200X and 400X) and microfilariae (mf) were counted.

*PCR* - The microfilariae DNA was extracted following the PROMEGA^®^ kit protocol (Promega, Madison, WI, USA). Identification of the filarial species was performed using nested PCR with two PCR reactions. The first reaction incorporated the primers rRNA2 and NC2, in 18S and 28S ribosomal DNA (rDNA), respectively, to amplify the ITS1-5.8S-ITS2 fragment from total nematode DNA and yield a 1097bp product (Nuchprayoon et al. 2005). The second reaction incorporated the products of the first reaction together with the primers FL1-F (5'-TTCCGTAGGTGAACCTGC-3') and Di660R (5'-ACCCTCAACCAGACGTAC-3') in 18S and 28S ribosomal DNA (rDNA), respectively, to yield a 552bp product which indicates the presence of *M. ozzardi*. Forward and reverse oligonucleotide primers were designed based on filarial parasite 18S and 5.8S rDNA conserved sequences, as previously described by Nuchprayoon et al. (2005). PCRs were performed in a 25 μL reaction mixture containing 20 mM Tris-HCl (pH 8.4), 1.5 mM MgCl_2_, 50 mM KCl, 0.2 mM of dNTPs, 1 unit of Taq DNA polymerase and 0.5 μM of each primer. The ITS1 forward primer (ITS1-F) was 5′-GGTGAACCTGCGGAAGGATC-3′ and the ITS1 reverse primer (ITS1-R) was 5′-GCGAATTGCAGACGCATTGAG-3′ (PCR reaction cycle: 94°C for 5 min; 40 cycles at 94°C for 30 s - 58°C for 30 s - 72°C for 90 s; and 7 min at 72°C). All oligonucleotide primers were obtained from the Alpha DNA (http://www.alphadna.com/contact.html Montreal, Quebec, Canada).

*Statistical analyses* - To construct the reference group (gold standard), the results of five diagnostics in a Bayesian latent class model (BLCM) were used. The BLCM is a statistical method for finding subgroups within a multivariate dataset. This method does not assume that the diagnosis of mansonelliasis can be confirmed by only one test. Instead, the diagnosis of mansonelliasis is determined by the pattern of association between the results of all five tests (Turecheck et al. 2013). We chose the model based on deviance criterion information (DIC), degrees of freedom (DF) and the maximum likelihood (ML). We used the best-fitted model to calculate the prevalence of mansonelliasis among subjects and calculated the sensitivity and specificity of all five tests. Furthermore, sensitivity, specificity and negative predictive value (NPV) also were calculated using membrane filtration as the gold standard. The BLCM was constructed using the BayesLCA package (White & Murphy 2014).

Logistic regression was used to compute the odds ratio (OR) and the probability of returning a positive result according to the quantity of microfilariae present in the sample. We performed all analyses on R 3.2.3 software (R Core Team 2015). All the models were submitted to residual analysis to check viability.

## RESULTS

From a total of 189 samples, the PCMF gold standard detected 67 samples (35.4%) that were positive for *M. ozzardi* microfilariae. PCR and Knott exhibited high sensitivity, detecting 66 (98.5%) and 56 (83.5%) positive samples, respectively. The DTBS and VTBS methods detected 51 (76.1%) and 47 (70.1%) positive samples, respectively. DTBS, VTBS and Knott showed a specificity of 100%, and there were no false negatives. PCR showed a specificity of 97.6%, indicating that there were false negatives.

Using BLCM we constructed two groups: (i) mansonelliasis and (ii) non-monsonelliasis. Both groups were constructed indirectly by the results of five diagnosis tests for mansonelliasis. According to these results, mansoneliasis prevalence in the samples was 30.1% (95% CI 26.6 - 33.6). When we use the mansonelliasis group to individually assess sensitivity, specificity and VPN of each test, we found that PCMF was the best test to identify a patient with mansonelliasis (100% sensitivity; 95% CI 93.7 - 100) followed by Knott (98.3%; 95% CI 90.6 - 99.9) and PCR (100%; 95% CI 93.7 - 100) (Table).

**TABLE:** Sensitivity, specificity and negative predictive values of the different diagnostic tests

		Membrane as gold standard % (95)	BLCM %(95)
Frequency of microfilaremics		35.4 (29.0 - 42.0)	30.1 (26.6 - 33.6)
Polymerase chain reaction			
	Sensitivity	98.5 (92.0 - 99.7)	100 (93.7 - 100)
	Specificity	97.6 (93.2 - 99.2)	91.7 (85.9 - 95.6)
	NPV	99.2 (95.5 - 99.8)	
Blood filtration in polycarbonate membrane			
	Sensitivity	-	100 (93.7 - 100)
	Specificity	-	100 (93.7 - 100)
	NPV		
Knott			
	Sensitivity	83.5% (72.9 - 90.5)	98.3 (90.6 - 99.9)
	Specificity	100% (96.9 - 100)	100 (97.2 - 100)
	NPV	91.7% (85.8 - 95.3)	
Digital thick blood smears			
	Sensitivity	76.1% (64.6 - 84.7)	89.5 (89.5 - 96.0)
	Specificity	100% (96.9 - 100)	100 (97.2 - 100)
	NPV	88.4% (82.1 - 92.7)	
Venous thick blood smears			
	Sensitivity	70.1% (58.3 - 79.8	82.5 (82.5 - 91.3)
	Specificity	100% (96.9 - 100)	100 (97.2 - 100)
	NPV	85.9% (79.2 - 90.7)	

*: tests used were digital thick blood smear (DTBS), venous thick blood smear (VTBS), polycarbonate membrane filtration (PCMF), Knott's method (Knott) and polymerase chain reaction (PCR);

**: PCMF was used as the gold standard. The results of the five diagnoses were analysed using the Baynesian latent class model (BLCM).

The probability of a positive result was altered by the quantity of microfilariae (quantified by PCMF) per mL of sample. The OR of returning a positive result by digital blood smear increased on average by a factor of 1.03 (95% CI 1.01 - 1.04) for an increase of one microfilariae per mL of sample. The same pattern was found for VTBS (1.02; 95% CI 1.01 - 1.03), Knott (1.0; 95% CI 1.03 - 1.08) and PCR (3.97; 95% CI 2.28 - 10.13).

**Fig. 2:**
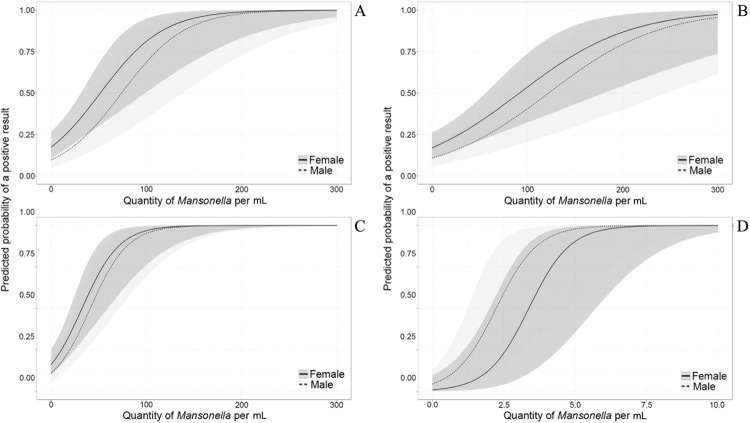
predicted probability of returning a positive result for *Mansonella ozzardi* in (A) digital thick blood smear, (B) venous thick blood smear, (C) Knott, and (D) polymerase chain reaction according to the quantity of microfilariae per mL quantified using polycarbonate membrane filtration of blood.

The quantity of microfilariae necessary to return a positive result was lower using PCR than for any other test: samples with more than eight microfilariae returned a positive result with 100% certainty. By contrast, when using DTBS, which is the most common diagnostic method, a sample with approximately 50 microfilariae per mL returned a positive result only 50% of the time, the equivalent of a coin toss (Fig. 2).

**Fig. 3:**
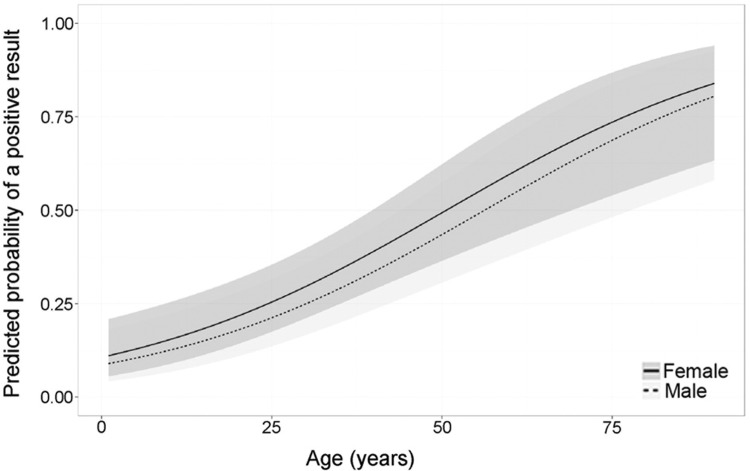
predicted probability and confidence interval of being infected by *Mansonella ozzardi* according to age and gender.

This pattern of infection and diagnosis exhibited no statistical difference between genders (p > 0.05). The gender-adjusted (gender-adjusted OR) of a positive diagnosis increased by 1.04 (1.02 - 1.06) for every year that a patient's age increased (Fig. 3).

## DISCUSSION

This is the first study of *M. ozzardi* in Brazil to use five diagnostic methods simultaneously and observe greater sensitivity in the PCMF, PCR and Knott methods. A few studies in Brazil have used more than one diagnostic method for identifying *M. ozzardi*. Moraes (1958) and Batista et al. (1960a) observed higher prevalence rates with the Knott diagnoses (41.4% and 56.7%, respectively) in relation to DTBS diagnoses (23.1% and 30.3%, respectively). It has been recently observed that PCR is 1.8 times more effective than DTBS for diagnosing the presence of *M. ozzardi* (Medeiros et al. 2015). There are also reports of low DTBS sensitivity in other countries. In Haiti, a higher prevalence of microfilaremics was detected by Knott (30.3%) in comparison to DTBS (24.1%) (Raccurt et al. 1982). In Venezuela, the PCMF and Knott prevalence rates were 41.2% and 36.7%, respectively, whereas DTBS exhibited far less sensitivity, detecting a prevalence rate of only 12.2% (Botto et al. 1983). The main diagnostic method used in the Amazon basin is DTBS. This study corroborates earlier findings that DTBS possesses less sensitivity than other diagnostic methods, and that the prevalence rates are therefore more likely to be underreported when DTBS is the method of diagnosis. Presently, the probability of underreporting prevalence was 1.35 (69/51) times lower for PCR, 1.31 (67/51) times lower for PCMF and 1.09 (56/51) times lower for Knott.

Microfilariaemia influences the probability that a given test will return a positive result. As the number of microfilariae in a sample increases the probability that a given test will return a positive result increases. Therefore, in regions where parasitized individuals have peripheral blood with low microfilariaemia, prevalence estimates are likely to be underreported when thick blood smears are the only method used for diagnosis. Moulia-Pelat et al. (1992) observed that the DTBS method detected only 44% (30/69) of microfilaremics with low and ultra-low microfilariae densities, and they concluded that DTBS was not reliable when a concentration method was used for diagnoses. Difference in sensitivity has also been reported in the diagnosis of *Wuchereria bancrofti*; the difference in sensitivity between thick blood smears and PCMF was small in patients with a microfilariae density above 33 mf/mL, but PCMF sensitivity was higher when microfilariaemia density was below 33 mf/mL (Kimura et al. 1984). In this study, DTBS detected only 66.7% of the microfilaremics detected by PCMF when samples had a density of 50 mf/mL or less.

The highest density of microfilariae was observed in subjects aged 30 - 45 and 45 - 60 years. Microfilariae may accumulate in older individuals because daily activities, such as farming and fishing, may expose these individuals to vectors more frequently and therefore lead to more frequent infection (Batista et al. 1960a, Medeiros et al. 2009, Martins et al. 2010). It is possible that a high degree of exposure could lead an individual to acquire the infection more than once, or to be parasitized by multiple females continuously producing microfilariae at high densities. An increase in microfilariae density with age was also observed by Botto et al. (1983), who recorded densities of 1.93 mf/mL in subjects 0 - 9 years of age and 192.3 mf/mL in subjects older than 50 years of age.

Most of the research on *M. ozzardi* conducted in Amazonas State, Brazil, has used thick blood smears for diagnosis. It is therefore highly likely that the prevalence rate of mansonelliasis has been underreported in several regions, especially regions where individuals exhibit low microfilariae density. This study addresses the problem of underreported infection by demonstrating that prevalence rates can be assessed more accurately using more sensitive diagnostic methods, such as Knott, PCMF and PCR.
